# Supplementary material for: Investigation of the molecular biology underlying the pronounced high gene targeting frequency at the Myh9 gene locus in mouse embryonic stem cells
Source: PLoS One. 2020 Mar 30;15(3):e0230126. doi: 10.1371/journal.pone.0230126 (PMC7105122; doi:10.1371/journal.pone.0230126)
Supplement: S1 Table — (DOCX) [file pone.0230126.s001.docx]

| **Primer** | **Sequence (5’-3’)** | **DNA band size** | **Usage** |
| --- | --- | --- | --- |
| **Primers used for creating targeting constructs** | | | |
| MA2L-F | ACGC**GTCGAC**GAAGTGAAGCTCCTGGCTTTG | 4.0kb | Amplifying the 5’ arm of the construct targeting to mouse Myh9 exon2 (MA2) |
| MA2L-R | TCC**CCGCGG**GTGACTTGCGGCCAGGACCTAAG |  |  |
| MA2R-F | GG**GGTACC**GGCTCAGCAGGCTGCAGACAAGTACCTC | 1.7kb | Amplifying the 3’ arm of the construct targeting to mouse Myh9 exon2 (MA2) |
| MA2R-R | CG**GGATCC**CAGCGGGGTAGGAAGCACGATG |  |  |
| MAI2L-F | ACGC**GTCGAC**GAAGTGAAGCTCCTGGCTTTG | 4.5kb | Amplifying the 5’ arm of the construct targeting to mouse Myh9 intron2 (MAI2) |
| MAI2L-R | TCC**CCGCGG**ctgcatgcagggaacagaggg |  |  |
| MAI2R-F | AGCTTT**GTTTAAAC**GAAGATCAAGCTCCCACCTGC | 2.0kb | Amplifying the 3’ arm of the construct targeting to mouse Myh9 intron2 (MAI2) |
| MAI2R-R | CG**GGATCC**GGAGGCTGAAGCCCTGCCCAG |  |  |
| MA3L-F | ACGC**GTCGAC**TCTAGATGAAGCCGATGCTGCATC | 4.3kb | Amplifying the 5’ arm of the construct targeting to mouse Myh9 exon3 (MA3) |
| MA3L-R | TCC**CCGCGG**ATCTCGTGCCTCTTCTTGCCCTTG |  |  |
| MA3R-F | AGCTTT**GTTTAAAC**CACATCTACGCCATCACAGATAC | 2.3kb | Amplifying the 3’ arm of the construct targeting to mouse Myh9 exon3 (MA3) |
| MA3R-R | CG**GGATCC**ATGCATCCTACAGTATCCCAAGTG |  |  |
| **Primers used for HR event identification** | | | |
| PE2I2E3-F | ATGAGGAAATTGCATCGCATTGTC | PE2I2E3-F+PE2-R=2.1kb | Identifying HR events occurring at Myh9 exon2 |
| PE2-R | TGGGACTCCTGGGTTGAGGGTTTTGG |  |  |
| PI2-R | GGAACCTCGATGCGCATACATAG | PE2I2E3-F+PI2-R=2.2kb | Identifying HR events occurring at Myh9 intron2 |
| PE3-R | GTCACAACTAGGGAGCATGAAAGAG | PE2I2E3-F+PE3-R=2.3kb | Identifying HR events occurring at Myh9 exon3 |
| **Primers used for creating truncated targeting constructs** | | | |
| LAP-F1 | ACGC**GTCGAC**GAAGTGAAGCTCCTGGCTTTG | LAP-F1+LAP-R=4.0kb LAP-F2+LAP-R=3.0kb  LAP-F3+LAP-R=2.0kb LAP-F4+LAP-R=1.0 | Amplifying the series of truncated left arms |
| LAP-F2 | ACGC**GTCGAC**TCATCATTTCCTGAGAGTCC |  |  |
| LAP-F3 | ACGC**GTCGAC**AAAGTGCTTAACTGCCCGTCC |  |  |
| LAP-F4 | ACGC**GTCGAC**TTCCTGAACTGCTTTCCCTC |  |  |
| LAP-R | **ATTT**GGTGACTTGCGGCCAGGACCTAAG |  |  |
| RAP-F | **GTTT**TGGCTCAGCAGGCTGCAGACAAGTACCTC | RAP-F+RAP-R1=1.7kb RAP-F+RAP-R2=1.1kb RAP-F+RAP-R3=0.6kp  RAP-F1+RAP-R1=1.2kb | Amplifying the series of truncated right arms |
| RAP-F1 | AGCTTT**GTTTAAAC**GAAGATCAAGCTCCCACCTGC |  |  |
| RAP-R1 | CG**GGATCC**CAGCGGGGTAGGTTGCACGATG |  |  |
| RAP-R2 | CG**GGATCC**AGGCATGGAGTTTGTGATGGAG |  |  |
| RAP-R3 | CG**GGATCC**TACAGGGTCTGACCAGGTGGTAC |  |  |

**Supplementary Table 1:** The PCR primers used in this study

Note: Underlined and bolded is the restriction site.
