# Supplementary material for: Investigation of the molecular biology underlying the pronounced high gene targeting frequency at the Myh9 gene locus in mouse embryonic stem cells
Source: PLoS One. 2020 Mar 30;15(3):e0230126. doi: 10.1371/journal.pone.0230126 (PMC7105122; doi:10.1371/journal.pone.0230126)
Supplement: Attachment — Submitted filename: Comments to the Author-PONE-D-19-15918.doc [file pone.0230126.s002.doc]

Comments to Authors
In this study, the authors intend to investigate the molecular biology underlying for the pronounced high gene targeting frequency at *Myh9* gene locus. Numerous factors which affect the HR efficiency were investigated, including chromosome position, transcriptional activity, targeted loci, and the length of the homology arm. Some issues should be addressed or discussed in detail below.

Major comments:
1. Authors constructed 5’ and 3’ homologous arm from the 129/Sv genomic BAC clone DNA, and then the mouse cells from hybrid C57BL/6 and 129S6/SvEv were targeted. For sequence divergence among laboratory mice and the divergence imposed a major handicap in mutating the gene through homologous recombination (see HEIN TE RIELE, 1992, ref#15), it is possible that the cells line background affects the HR efficiency. The authors should sequence the target locus of cell lines to ensure that is consistent with the homologous arm sequences.

2. Authors declared that “No animal experiments were conducted, no ethic permits were therefore needed for this report”, however, “MEFs were isolated and cultured embryonic day 9.5 to 10.5 (line 130, ref#29)”.

3. The authors did analyze the locus of the *Myh9* gene in multiple species. However, there is no data showing the GT frequency in these species. Therefore, it’s hardly to reach the conclusion that low correlation with the high GT frequency at the *Myh9* gene in term of chromosome position, from the variable or conserved the locus *Myh9* and its flanking genes. And several other types of genomic elements should be analyzed which may affect GT efficiency in *Myh9*, including CpG islands, simple repeats, microsatellites, DNA transposons, SINEs, LINEs *et al.*

4. The authors declared that the length and ratio of homologous arms affected the GT efficiency (Table 4). However, when targeting the *Myh9* gene Exon 2, Intron 2 and Exon 3, why choose similar length of the homologous arms rather than the same length? And Dose the difference length and ratio of homologous arms also lead to the results?

Minor comments:

5. In Figure 1A, The Unch is short for? In Figure 1B shows the Divergence but the Figure legend is similarity.

6. In Figure 2A, what does the black triangle indicates? In Figure 2B and 2D, the mRNA level and the protein level of NMHC IIA in ES cells were set as 100%, the SD should not exist in ES cells columns.

7. Authors should provide the data of identify recombination events

8. The antibodies company should be labeled.

9. Many grammatical or spelling should be corrected thoroughly in the manuscript.
