# Supplementary material for: Investigation of the molecular biology underlying the pronounced high gene targeting frequency at the Myh9 gene locus in mouse embryonic stem cells
Source: PLoS One. 2020 Mar 30;15(3):e0230126. doi: 10.1371/journal.pone.0230126 (PMC7105122; doi:10.1371/journal.pone.0230126)
Supplement: Attachment — Submitted filename: Response to Reviewers.docx [file pone.0230126.s003.docx]

**Response to Reviewers**

**Reviewer #1**: In the manuscript entitled “Investigation of the molecular biology underlying the pronounced high gene targeting frequency at Myh9 gene locus in mouse embryonic stem cells”, the authors perform homologous recombination (HR) in mouse embryonic stem cells (ESCs), induced pluripotent stem cells (iPSCs), and embryonic fibroblasts (MEFs) to investigate the molecular features of the Myh9 gene locus that are responsible for the notably high efficiency of gene targeting. They perform comparative genetic sequence analysis of the Myh9 gene locus to illustrate a low degree of sequence conservation, which in turn suggests there are no unique genetic properties at these loci that affect recombination efficiency. By targeting their vector into the exon 2 region of Myh9 in mouse ESCs, iPSCs, and MEFs, the authors demonstrate a variable HR efficiency that is cell type-dependent but transcriptionally and translationally independent. The efficiency of HR also declines as the targeting vector is shifted downstream of exon 2, highlighting the contributions of local regions to recombination efficiency. Finally, the authors investigate the effects of modifying the lengths of the homology arms and discover an optimal ratio of 5’ to 3’ arm lengths for effective HR. Because gene editing has proven itself as an indispensable tool in biomedical research, primarily in enabling the generation of genetic models of development and disease, it is a worthwhile effort to understand the mechanisms regulating these processes. Despite the enormous strides in gene editing technology over the past decade using programmable nucleases, challenges remain in the field such as increasing the size of the targeting vector or minimizing off target genetic modifications. Understanding the properties of cis and trans factors affecting the efficiency of gene targeting through classical HR can yield mechanistic insights that have positive implications for other gene targeting technologies. While to goal of this manuscript is to advance the mechanistic understanding of how properties of the Myh9 locus enable such high gene targeting efficiencies through HR, the low experimental robustness and interpretation presented in this manuscript limit the validity of the conclusions drawn. It is by these qualities that this manuscript as submitted is not appropriate for publication in PLOS One, and descriptions of these qualities are outlined below.

**Answer: All of the authors thank the reviewer for the comments on this manuscript. In the revised version, we perform more detailed bioinformatic analysis, remove the not well addressed part conducted in mESCs, miPSCs and MEFs, and also provide necessary results. Moreover, we answer the concerns/questions raised by the reviewer in a point-to-point way as following.**

**Question #1:** **A:** First, it is unclear in the written text what the authors aim to convey from the sequence comparisons across different species. **B:** Is there prior evidence that sequence conservation predicts strong recombination effects? **C:** Is there evidence that HR is high or low in ESCs of other species? If, for instance, HR efficiency is comparable in both rat and mouse ESCs (PMID: 20703227, PMID: 21151976), can conserved sequences be found across both loci? **D:** If there are discrepancies in HR efficiency across two species despite conserved sequences within syntenic elements, this could suggest differential epigenetic effectors. The authors can incorporate efficiency data from other ESC studies and better frame the interpretations drawn from their analysis. **E:** Furthermore, the data presented in Table 1, specifically the percent sequence similarity among the homology arms should be analyzed separately for the 5’ and 3’ arms. Figure 1A, or any of the other gene diagrams are not to scale and should indicate this.

**Answer #1: We thank the reviewer for the comments. The questions in this paragraph are split into multiple ones and answered separately.**

**A: To our knowledge, this is the first time that such a high homologous recombination efficiency was reported and extensively investigated in mammalian cells based on traditional homologous recombination-mediated gene targeting [Liu T et al, PLoS One. 2018]. This property has facilitated the generation of a series of mouse models [Wang A et al, Proc Natl Acad Sci U S A. 2010; Zhang Y et al, Blood. 2012]. This efficiency is even much higher than using much longer homologous arms, e.g bacterial artificial chromosomes (BAC) DNA (6%,** [**Testa G**](https://www.ncbi.nlm.nih.gov/pubmed/?term=Testa%20G%5BAuthor%5D&cauthor=true&cauthor_uid=12627172) **et al.** [**Nat Biotechnol.**](https://www.ncbi.nlm.nih.gov/pubmed/12627172) **2003, 21(4):443-7), or designer nucleases-facilitated (ZFN, TALEN, and CRISPR) gene-targeting (≥20%,** [**Casola S**](https://www.ncbi.nlm.nih.gov/pubmed/?term=Casola%20S%5BAuthor%5D&cauthor=true&cauthor_uid=20827532)**.** [**Methods Mol Biol.**](https://www.ncbi.nlm.nih.gov/pubmed/20827532) **2010, 667:145-63). We have consulted a number of experts who study homologous recombination, but have not gained any useful clues on investigating the underlying mechanisms for this unique phenomenon. HR efficiency is collectively decided by many factors such as the target locus itself, isogenic DNA, length of homologous arms, transcription activity of the target locus, and the status of the ES cells [**[**Ledermann B**](https://www.ncbi.nlm.nih.gov/pubmed/?term=Ledermann%20B%5BAuthor%5D&cauthor=true&cauthor_uid=11187956)**,** [**Exp Physiol.**](https://www.ncbi.nlm.nih.gov/pubmed/?term=Experimental+Physiology+(2000)+85.6%2C+603-613) **2000, 85(6):603-13]. Additionally, previous study indicated that meiotic recombination hotspots in mice are clustered with the major histocompatibility complex (MHC) region (**[**Paul P**](https://www.ncbi.nlm.nih.gov/pubmed/?term=Paul%20P%5BAuthor%5D&cauthor=true&cauthor_uid=26991854) **et al.** [**DNA Repair**](https://www.ncbi.nlm.nih.gov/pubmed/26991854)**. 2016,** **40:47-56), implying the genetic context matters. Meanwhile, it was suggested that comparative analysis of DNA sequences from multiple species at varying evolutionary distances is a powerful approach for identifying sequences that are unique for a given organism [Frazer KA, et al. Genome Res. 2003;13(1):1-12]. As an initial effort, we compared the genes flanking the Myh9 locus and the Myh9 sequences of mouse used to generate the homology arms with those of other species. The aim of these comparisons is to examine whether the Myh9 gene locus has undescribed and unique properties among different species including the conservation in terms of genetic context and whether there are distinct cis-elements, resembling LoxP, FRT, and other sequences, contributing to this high HR efficiency. The locus and sequence comparison could at least rule out this possibility or provide clues.**

**B: There is no evidence indicating that sequence conservation predicts higher recombination rate, but there are indications that sequence divergence has an inhibitory effect on homologous recombination [[Lukacsovich T](https://www.ncbi.nlm.nih.gov/pubmed/?term=Lukacsovich%20T%5BAuthor%5D&cauthor=true&cauthor_uid=10101177) et al.** [**Genetics.**](https://www.ncbi.nlm.nih.gov/pubmed/?term=Suppression+of+Intrachromosomal+Gene+Conversion+in+Mammalian+Cells+by+Small+Degrees+of+Sequence+Divergence) **1999, 57(1):43-56;** [**Opperman R**](https://www.ncbi.nlm.nih.gov/pubmed/?term=Opperman%20R%5BAuthor%5D&cauthor=true&cauthor_uid=15611187) **et al.** [**Genetics.**](https://www.ncbi.nlm.nih.gov/pubmed/15611187) **2004,** **168(4):2207-15].**

**C: Mouse is the only species, in which ES cells have been extensively used in gene-targeting experiments. Germline-competent ES cells have also been generated in rats, but very few gene-targeting experiments have been reported. ES cell lines from some other mammalian species have been reported, but they have not been proven to be able to transmit through the germline, and therefore not used much in gene knockout experiment. No reports or data are available for comparing the targeting efficiency in mouse and rat embryonic stem cells. In general, the ES cells-based gene targeting efficiency is around 1-10% in the species of mouse [**[**Brown AC**](https://www.ncbi.nlm.nih.gov/pubmed/?term=Brown%20AC%5BAuthor%5D&cauthor=true&cauthor_uid=19002898) **et al.** [**Cytotechnology.**](https://www.ncbi.nlm.nih.gov/pubmed/?term=Pooling+and+PCR+as+a+method+to+combat+low+frequency+gene+targeting+in+mouse+embryonic+stem+cells) **2006,** **51(2):81-8]. As far as the cases mentioned by the reviewer, it seems the HR efficiencies in those two cell types are comparable without considering the strategies and conditions used [[Doetschman T](https://www.ncbi.nlm.nih.gov/pubmed/?term=Doetschman%20T%5BAuthor%5D&cauthor=true&cauthor_uid=3186749) et al.** [**Proc Natl Acad Sci U S A.**](https://www.ncbi.nlm.nih.gov/pubmed/3186749) **1988, 85(22):8583-7;** [**Donehower LA**](https://www.ncbi.nlm.nih.gov/pubmed/?term=Donehower%20LA%5BAuthor%5D&cauthor=true&cauthor_uid=1552940) **et al.** [**Nature.**](https://www.ncbi.nlm.nih.gov/pubmed/1552940) **1992, 356(6366):215-21;** [**Meek S**](https://www.ncbi.nlm.nih.gov/pubmed/?term=Meek%20S%5BAuthor%5D&cauthor=true&cauthor_uid=21151976) **et al.** [**PLoS One.**](https://www.ncbi.nlm.nih.gov/pubmed/?term=PMID%3A+21151976) **20105(12):e14225; Tong C et al. Nature. 2010, 467(7312):211-3], in which a targeting efficiency of 1-3.7% can be achieved in the ES cells from both species.**

**D: It is worth mentioning that most targeting efficiencies reported in the literature are within an expected range, together with multiple determinants for the efficiency, make it difficult to compare these cases. Likewise, a tight and direct relationship between epigenetic factors and homologous recombination(HR)-based gene targeting remains lacking, although there are a lot of studies indicating the contribution of these epigenetic factors to HR-directed DNA repair [[Vélez-Cruz R](https://www.ncbi.nlm.nih.gov/pubmed/?term=V%C3%A9lez-Cruz%20R%5BAuthor%5D&cauthor=true&cauthor_uid=27940962) et al.** [**Genes Dev.**](https://www.ncbi.nlm.nih.gov/pubmed/27940962) **2016, 30(22):2500-2512;** [**Lang F**](https://www.ncbi.nlm.nih.gov/pubmed/?term=Lang%20F%5BAuthor%5D&cauthor=true&cauthor_uid=28973861) **et al.** [**Proc Natl Acad Sci U S A.**](https://www.ncbi.nlm.nih.gov/pubmed/28973861) **2017, 114(41):10912-10917;** [**Chakraborty U**](https://www.ncbi.nlm.nih.gov/pubmed/?term=Chakraborty%20U%5BAuthor%5D&cauthor=true&cauthor_uid=31221666) **et al.** [**Genetics.**](https://www.ncbi.nlm.nih.gov/pubmed/31221666) **2019, 212(4):1147-1162]. Undoubtedly, the property of high gene targeting frequency at the MyH9 gene locus reported here facilitates to investigate those interesting questions in the future.**

**E: Additionally, the bioinformatic analysis of the similarity among the homology arms is further conducted and presented, and the claim of the gene diagrams not to scale is added in the revised version.**

**Question #2:** **A:** Next, the authors should report on how many different ESC, iPSC, and MEF clonal lines were used to produce the data presented in Figure 2, as well as what passage number. If a single clonal cell line were used for each, this limits the generalizability of their findings. Furthermore, if these properties change over prolonged passaging of cell lines, this could also explain effects on efficiency. **B:** An ideal experiment to truly compare epigenetic states would be to conduct HR experiments on cell types derived from the same genetic individual. For instance, the authors could in vitro differentiate an ESC line into somatic cells such as fibroblasts, then reprogram them into iPSCs, and perform HR experiments on all three epigenetic states. **C:** Methodologically, the PCR should be designed in a way to detect a PCR product produced from the wild type alleles, as opposed to seeing no PCR product amplified. When no PCR band is detected for that sample, it could have resulted from a failed PCR run. The agarose gel images should be presented. Additionally, the authors should indicate where and whether the qPCR primers and antibody epitope account for exon2.

**Answer #2: We thank the reviewer for the comment. A: Since the similar concern was raised by all reviewers, we remove the part involving the experiment conducted in miPSCs and MEFS after communicating with the Academic editor, Dr Hu. All mouse ESC gene-targeting experiments were conducted using passage 14 (P14) V6.5 ES cell line. Our transgenic core has frozen hundreds of vials of P14 V6.5 ES cells, and we thaw a new vial for each construct. Therefore, all constructs were done using the same passage cells and the cell cycle/proliferation rate should be very similar. B: miPSC and MEF data have been removed. C: The long-range PCR using one primer located at the Neomycin selection maker and one primer resided in the outside of the short arm, has widely been used to identify the gene targeting recombinants in our previous and other studies [Liu T et al, PLoS One. 2018, 13(2):e0192641; Zhou D et al. J Vis Exp. 2018, (141);** [**Lay JM**](https://www.ncbi.nlm.nih.gov/pubmed/?term=Lay%20JM%5BAuthor%5D&cauthor=true&cauthor_uid=9608741) **et al.** [**Transgenic Res.**](https://www.ncbi.nlm.nih.gov/pubmed/9608741) **1998, 7(2):135-40;** [**Anastassiadis K**](https://www.ncbi.nlm.nih.gov/pubmed/?term=Anastassiadis%20K%5BAuthor%5D&cauthor=true&cauthor_uid=24182921) **et al.** [**Methods Enzymol.**](https://www.ncbi.nlm.nih.gov/pubmed/24182921) **2013533:133-55;** [**Fisher CL**](https://www.ncbi.nlm.nih.gov/pubmed/?term=Fisher%20CL%5BAuthor%5D&cauthor=true&cauthor_uid=28981838) **et al.** [**Nucleic Acids Res.**](https://www.ncbi.nlm.nih.gov/pubmed/?term=An+efficient+method+for+generation+of+bi-allelic+null+mutant+mouse+embryonic+stem+cells+and+its+application+for+investigating+epigenetic+modifiers) **2017, 45(21):e174; Sommer D et al. Nat Commun. 2014, 5:3045]. Furthermore, a positive and a negative control were included in each run of PCR to rule out the PCR failure. The PCR products were also sequenced to further confirm the targeting events. Additionally, representative agarose gel images are presented in the revised version, all the PCR primers have been listed in the supplementary table 1.**

**Question #3:** **A:** Lastly, what is the contribution of cell cycle of each cell type to the HR efficiency? Could a difference in cell cycling rate between the ESC and iPSC lines examined explain the difference in the rate of successful targeting? **B:** In sum, this manuscript falls short of the technical standards and logical conclusions expected from PLOS publications. Until the authors address the shortcomings described above and write a coherent story based on tenable data, this work should not be published as presented.

**Answer #3: We thank the reviewer for the comment. A: As stated above, we have removed the experiments conducted in miPSCs and MEFs per the permission of the academic editor, Dr. Hu, though our observation of the gene targeting efficiency in mouse ES cells higher than that in iPS cells is to some degree consistent with a previous study [**[**Zou J**](https://www.ncbi.nlm.nih.gov/pubmed/?term=Zou%20J%5BAuthor%5D&cauthor=true&cauthor_uid=19540188) **et al.** [**Cell Stem Cell.**](https://www.ncbi.nlm.nih.gov/pubmed/?term=Gene+Targeting+of+a+Disease-Related+Gene+in+Human+Induced+Pluripotent+Stem+and+Embryonic+Stem+Cells) **2009, 5(1):97-110]. B: We believe that our revised version has been improved and should reach the standard of the journal and the response to the comments facilitates to understand the story of this manuscript.**

**Reviewer #2**: Tan and co-workers explore the efficiency of traditional gene targeting of the murine Myh9 locus. The work in the manuscript is focused on the mouse Myh9 locus, however, the discussion includes speculation about other species with relatively shallow bioinformatics analysis. Conversely, detailed analysis of the murine Myh9 locus and the sequence of the homology arms lacks any sizable detail. While the identification of the murine Myh9 locus as a mitotic recombination hotspot would be of interest for the field of genome editing, especially for those trying to optimize gene targeting, the manuscript as is lacks any in depth analysis. The manuscript is both highly speculative about the underlying cause of the high efficiency targeting, while being relatively shallow about the details of the actual molecular work that was done. The manuscript would be greatly improved if it was focused on the mouse Myh9 locus and provided greater details about the various targeting vectors utilized and the genome editing efficiencies observed.

**Answer: All of the authors thank the reviewer for the comments on this manuscript. In the revised version, we perform more detailed bioinformatic analysis, remove the not well addressed part conducted in mESCs, miPSCs and MEFs, and also provide necessary results. Moreover, we answer the concerns/questions raised by the reviewer in a point-to-point way as following.**

Major Comments

**Question #1):** The comparison of the Myh9 locus in various species does not provide any insight into the high targeting efficiency of the murine Myh9 locus. In this manuscript, only the mouse genome is targeted, and it is inappropriate to infer anything about the targeting potential of the Myh9 locus in these other species, and the contribution of the flanking genomic sequence and the species-specific Myh9 locus. Unless IPSCs from these various species are also used for homologous gene targeting of the Myh9 locus, there is little information to be gained from comparing the Myh9 loci.

**Answer #1: The authors thank the reviewer for this suggestion. 1) it was suggested that comparative analysis of DNA sequences from multiple species at varying evolutionary distances is a powerful approach for identifying sequences that are unique for a given organism [Frazer KA, et al. Genome Res. 2003;13(1):1-12]. 2) The aim of these comparisons is to examine whether the Myh9 gene locus has undescribed and unique properties among different species including the conservation in terms of genetic context and whether there exist unique elements like LoxP element contributing to this high HR efficiency. The locus and sequence comparison could at least rule out this possibility or provide other clues. 3) To our knowledge, it is the first time to report a so high homologous recombination efficiency at the Myh9 gene locus in mouse embryonic stem cells based on traditional gene targeting [Liu T et al, PLoS One. 2018], this property has facilitated the generation of a series of mouse models [Wang A et al, Proc Natl Acad Sci U S A. 2010; Zhang Y et al, Blood. 2012]. This efficiency is also much higher than that of even using longer homology arms, e.g bacterial artificial chromosomes (BAC) DNA (6%,** [**Testa G**](https://www.ncbi.nlm.nih.gov/pubmed/?term=Testa%20G%5BAuthor%5D&cauthor=true&cauthor_uid=12627172) **et al.** [**Nat Biotechnol.**](https://www.ncbi.nlm.nih.gov/pubmed/12627172) **2003, 21(4):443-7) and that of widely used safe harbor site ROSA26 in mouse embryonic stem cells (≥20%,** [**Casola S**](https://www.ncbi.nlm.nih.gov/pubmed/?term=Casola%20S%5BAuthor%5D&cauthor=true&cauthor_uid=20827532)**.** [**Methods Mol Biol.**](https://www.ncbi.nlm.nih.gov/pubmed/20827532) **2010, 667:145-63). We have even consulted with several experts in the field. Frankly to say, we could not get useful clues to investigate the underlying mechanisms for this unique phenomenon. HR efficiency is collectively decided by many factors such as the target locus itself, isogenic DNA, length of homologous arms, transcription activity of the target locus, and the status of the ES cells [**[**Ledermann B**](https://www.ncbi.nlm.nih.gov/pubmed/?term=Ledermann%20B%5BAuthor%5D&cauthor=true&cauthor_uid=11187956)**,** [**Exp Physiol.**](https://www.ncbi.nlm.nih.gov/pubmed/?term=Experimental+Physiology+(2000)+85.6%2C+603-613) **2000, 85(6):603-13]. Additionally, previous study indicated that meiotic recombination hotspots in mice are clustered with the major histocompatibility complex (MHC) region (**[**Paul P**](https://www.ncbi.nlm.nih.gov/pubmed/?term=Paul%20P%5BAuthor%5D&cauthor=true&cauthor_uid=26991854) **et al.** [**DNA Repair**](https://www.ncbi.nlm.nih.gov/pubmed/26991854)**. 2016,** **40:47-56), implying the genetic context matters. As the initial effort, we compared the genes flanking the Myh9 locus and the mouse Myh9 sequences used to generate the homology arms with those of other species. 4) The detailed comparisons revealed that the mouse Myh9 gene locus is not unique in terms of genetic context including its flanking genes and gene length, while the sequences used to create the homology arms have higher similarity with that of Rat than with those of other species, moreover, the homology of the right arm sequence is higher than that of the left one. Additionally, the right arm is GC rich relative to the left arm and those from other species. Further investigations are warranted to determine whether these properties are associated with the GT efficiency.**

**Question #2):** Although many aspects of homologous directed targeting remain unknown, it is accepted that critical for homologous targeting is mitosis and proliferation. Therefore, the inability to target the Myh9 locus in MEFs, which have a lower proliferative index and readily senescence in culture, is not surprising. The lack of senescence and high proliferation rate of ESCs and IPSCs is critical for efficient homologous gene targeting. Therefore, the expression level of Myh9 in MEFs is inconsequential in this reviewer’s opinion. The proliferation rate/index of the IPSCs and ESCs used in this study should be compared.

**Answer #2: The authors thank the reviewer for this concern. Indeed, the cell cycle is an important factor affecting GT efficiency [**[**Majumdar A**](https://www.ncbi.nlm.nih.gov/pubmed/?term=Majumdar%20A%5BAuthor%5D&cauthor=true&cauthor_uid=12538585) **et al.** [**J Biol Chem.**](https://www.ncbi.nlm.nih.gov/pubmed/?term=Cell+Cycle+Modulation+of+Gene+Targeting+by+a+Triple+Helix-forming+Oligonucleotide) **2003, 278(13):11072-7]. Since the point could not well addressed in the study, we remove the part involving the experiment conducted in miPSCs and MEFS after communicating with the Academic editor, Dr Hu.**

**Question #3):** With gene targeting efficiency over 50%, it would be expected that some of the clones to be homozygous for the targeted allele. For a locus specific report about homologous targeting, zygosity of targeting should be added to the manuscript.

**Answer #3: The V6.5 ESCs were derived from F1 hybrid mice, with 50% 129 and 50% C57BL/6, while the targeting constructs were made using 129 genomic DNA. Therefore, the constructs are isogenic to one of the two chromosomes. Importantly, targeting only one chromosome is advantageous in this case because homozygous knockout of Myh9 can cause abnormal proliferation and ESC morphology [Conti MA et al. J Biol Chem. 2004;279(40):41263-6], which can result in bias for colony picking. No ES clones with double knockout alleles were identified whether using PCR or Southern Blot methods [Wang A et al. Proc Natl Acad Sci U S A. 2010;107(33):14645-50; Zhang Y et al. Blood. 2012;119(1):238-50; Liu T et al. PLoS One. 2018;13(2):e0192641]. This information has been added in the revised version.**

**Question #4):** The data presented in Table 4 has a good linear relationship between total homology length and targeting efficiency. As presented in the table, it is difficult to see that relationship.

**Answer #4: Thanks for the useful suggestion. To better indicate the relationship between total length of homology arms and GT efficiency, we have reorganized the data and provided more detailed analysis. Indeed, there exists a linear relationship between those two, as shown in Figure 3 in the revised version.**

Question #5): The varying targeting efficiency between the three regions of the murine Myh9 locus discussed in Table 3 warrant some additional sequence analysis, such as GC content, repetitive elements, homology to other location in the murine genome, etc.

**Answer #5: Thank the reviewer for this good suggestion. We performed further sequence analysis of the constructs targeting to different sites of the Myh9 gene locus, including GC content, CpG island, simple sequence repeat (SSR), SINE, LINE, or other DNA elements, as summarized in the table 3 in the revised version. Interestingly, there is a consistency between the percentage of SSR and SINE and the efficiency of GT. No significant similarity between the sequences used to create these targeting constructs and the sequences of other regions in the mouse genome was observed.**

**Question #6):** Was chromatin accessibility assessed for the Myh9 locus? Even if not in the actual ESC line used, it would be interesting to know the status of the chromatin/nucleosome in mESCs of the readily targetable Myh9 locus.

**Answer #6: Thank the reviewer for the comment. Frankly to say, the information about the status of the chromatin accessibility at the Myh9 locus in mESCs could not facilitate to explain the readily targetable Myh9 locus. On one hand, it has been suggested that the chromatin state influences the targeting or editing efficiency mediated by Triplex-forming oligonucleotides (TFOs) or genome editing tools [Macris MA et al. J Biol Chem. 2003 Jan 31;278(5):3357-62;** [**van Rensburg R**](https://www.ncbi.nlm.nih.gov/pubmed/?term=van%20Rensburg%20R%5BAuthor%5D&cauthor=true&cauthor_uid=22436965) **et al.** [**Gene Ther.**](https://www.ncbi.nlm.nih.gov/pubmed/?term=Chromatin+structure+of+two+genomic+sites+for+targeted+transgene+integration+in+induced+pluripotent+stem+cells+and+hematopoietic+stem+cells) **2013 Feb;20(2):201-14;** [**Daboussi F**](https://www.ncbi.nlm.nih.gov/pubmed/?term=Daboussi%20F%5BAuthor%5D&cauthor=true&cauthor_uid=22467209) **et al.** [**Nucleic Acids Res.**](https://www.ncbi.nlm.nih.gov/pubmed/22467209) **2012 Jul;40(13):6367-79], while no evidence indicate the effect of chromatin accessibility on traditional HR-based gene targeting. On the other hand, earlier studies demonstrated that GT frequency is independent of target gene transcription [Johnson RS et al. Science. 1989,245(4923):1234-6;** [**Yáñez RJ**](https://www.ncbi.nlm.nih.gov/pubmed/?term=Y%C3%A1%C3%B1ez%20RJ%5BAuthor%5D&cauthor=true&cauthor_uid=9578833) **et al.** [**Gene Ther.**](https://www.ncbi.nlm.nih.gov/pubmed/9578833) **1998, 5(2):149-59]. More importantly, our previous studies indicated that the expression level of the Myh9 gene in mESCs is considerable [Conti MA et al. J Biol Chem. 2004,279(40):41263-6; Wang A et al. Proc Natl Acad Sci U S A. 2010;107(33):14645-50; Zhang Y et al. Blood. 2012;119(1):238-50; Liu T et al. PLoS One. 2018;13(2):e0192641].**

Minor Comments
1) Minor grammatical errors such as line 64: “…is rarely”

**A: This error has been corrected. Furthermore, we have also corrected some other grammatical errors during revision.**

2) Line 142/143; the purpose of the sub-cloning is unclear.

**A: The purpose of cloning PCR products into T-easy for sequencing is to make sure the GT recombinants identified by PCR are absolutely right ones. Because one primer is located at the 3’end of Neomycin and another one resides outside of the short arm, the sequencing result can confirm this.**

3) The choice of using the 129-derived BAC DNA as a template for the homology arms and the use of B6129F1 ES cells for electroporation is unclear given that isogenic homology arms are believed to increase targeting efficiency (Line 68).

**A: The V6.5 ESCs were derived from F1 hybrid mice, with 50% 129 and 50% C57BL/6, while the targeting constructs were made using 129 genomic DNA. Therefore, the constructs are isogenic to one of the two chromosomes. Importantly, targeting only one chromosome is advantageous in this case because homozygous knockout of Myh9 can cause abnormal proliferation and ESC morphology [Conti MA et al. J Biol Chem. 2004;279(40):41263-6], which can result in bias for colony picking.**

4) If available, the targeting efficiency of the Myh9 locus in the absence of negative selection (ganciclovir) would be a useful comparison, since negative selection in practice rarely results in the enrichment of correctly targeted clones that are conceptually expected.

**A: We had no data for GT efficiency at the Myh9 gene locus in the absence of ganciclovir.**

5) Source/method of IPSCs generation not indicated.

**A: After communicating with the academic editor Dr. Hu, we decided to remove the part involving the experiments conducted in miPSCs and MEFs, thus, the source/method of iPSCs generation is no longer needed.**

**Reviewer #3**: In this study, the authors intend to investigate the molecular biology underlying for the pronounced high gene targeting frequency at Myh9 gene locus. Numerous factors which affect the HR efficiency were investigated, including chromosome position, transcriptional activity, targeted loci, and the length of the homology arm. Some issues should be addressed or discussed in detail below.

**A: The authors thank the reviewer for providing comments on this manuscript. The concerns or issues are addressed below and in the revised version.**

Major comments:

1. Authors constructed 5’ and 3’ homologous arm from the 129/Sv genomic BAC clone DNA, and then the mouse cells from hybrid C57BL/6 and 129S6/SvEv were targeted. For sequence divergence among laboratory mice and the divergence imposed a major handicap in mutating the gene through homologous recombination (see HEIN TE RIELE, 1992, ref#15), it is possible that the cells line background affects the HR efficiency. The authors should sequence the target locus of cell lines to ensure that is consistent with the homologous arm sequences.

**Answer #1: Thanks for this comment. The V6.5 ESCs were derived from F1 hybrid mice, with 50% 129 and 50% C57BL/6, while the targeting constructs were made using 129 genomic DNA. Therefore, the constructs are isogenic to one of the two chromosomes. Importantly, targeting only one chromosome is advantageous at least in this case because homozygous knockout of Myh9 can cause abnormal proliferation and ESC morphology [Conti MA et al. J Biol Chem. 2004;279(40):41263-6], which can result in bias for colony picking. Additionally, no ES clones with double alleles knocked out were identified whether using PCR or Southern Blot method.**

2. Authors declared that “No animal experiments were conducted, no ethic permits were therefore needed for this report”, however, “MEFs were isolated and cultured embryonic day 9.5 to 10.5 (line 130, ref#29)”.

**Answer #2: Thanks for reminding this point. We are sorry for not paying attention to it. After communicating with the academic editor Dr Hu, we decide to remove the part involving the experiments conducted miPSCs and MEFs, thus, the source/method of MEFs generation should be removed too. Thus, no animal experiments are indeed involved.**

3. The authors did analyze the locus of the *Myh9* gene in multiple species. However, there is no data showing the GT frequency in these species. Therefore, it’s hardly to reach the conclusion that low correlation with the high GT frequency at the *Myh9* gene in term of chromosome position from the variable or conserved the locus *Myh9* and its flanking genes. And several other types of genomic elements should be analyzed which may affect GT efficiency in *Myh9*, including CpG islands, simple repeats, microsatellites, DNA transposons, SINEs, LINEs *et al.*

**Answer #3: Thanks for this good suggestion. According to the suggestion, we have performed further bioinformatic analysis on the sequences used to create construct targeting to the Myh9 gene exon2 in the mouse genome and the corresponding sequences from other species, the results are summarized and presented in the revised version.**

4. The authors declared that the length and ratio of homologous arms affected the GT efficiency (Table 4). However, when targeting the *Myh9* gene Exon 2, Intron 2 and Exon 3, why choose similar length of the homologous arms rather than the same length? And Dose the difference length and ratio of homologous arms also lead to the results?

**Answer #4: Thanks for this inquiry. Firstly, a 11.5kb region spanning from the upstream of exon2 to the downstream of the exon3 was used to create those three targeting constructs. These constructs were made at different times. Due to the availability of restriction sites and PCR primer sequences, sometimes it is hard to make constructs with exact the same length. The GT efficiency is determined by multiple factors, such as the locus, the length of the homologous arms [**[**Ledermann B**](https://www.ncbi.nlm.nih.gov/pubmed/?term=Ledermann%20B%5BAuthor%5D&cauthor=true&cauthor_uid=11187956)**,** [**Exp Physiol.**](https://www.ncbi.nlm.nih.gov/pubmed/?term=Experimental+Physiology+(2000)+85.6%2C+603-613) **2000, 85(6):603-13]. Thirdly, according to previous studies and our observation, the length and ratio of the homologous arms influences the GT efficiency. In current case, the length and ratio of the homologous arms of the constructs targeting to the Myh9 exon2, intron2 and exon3 was slightly increased, which were done unintentionally. Surprisingly, the GT efficiency is contrary to the length and ratio of the homologous arms (Figure 2 and table 2), suggesting other factors are responsible for this.**

Minor comments:

5. In Figure 1A, The Unch is short for? In Figure 1B shows the Divergence but the Figure legend is similarity.

**Answer #5: Thanks for the inquiry and correction. The Unch is short for uncharacterized, as indicated in the figure legend. In Figure1B, both the percentage of divergence and similarity are indicated.**

6. In Figure 2A, what does the black triangle indicates? In Figure 2B and 2D, the mRNA level and the protein level of NMHC IIA in ES cells were set as 100%, the SD should not exist in ES cells columns.

**Answer #6: Thanks for the inquiry. The part involving the experiments conducted in mESCs, miPSCs and MEFs has been removed from the manuscript after communicating with the academic editor Dr Hu.**

7. Authors should provide the data of identify recombination events

**Answer #7: Thanks for the suggestion. Representative images of PCR identification of HR events were provided in the revised version.**

8. The antibodies company should be labeled.

**Answer #8: Thanks for the suggestion, but it is unnecessary since that part has been removed.**

9. Many grammatical or spelling should be corrected thoroughly in the manuscript.

**Answer #9: Thanks for this suggestion. The manuscript has been revised and the grammatical or spelling errors has been checked by a native speaker Dr Adelstein (NIH/NHLBI).**
