# Supplementary material for: Investigation of the molecular biology underlying the pronounced high gene targeting frequency at the Myh9 gene locus in mouse embryonic stem cells
Source: PLoS One. 2020 Mar 30;15(3):e0230126. doi: 10.1371/journal.pone.0230126 (PMC7105122; doi:10.1371/journal.pone.0230126)
Supplement: Attachment — Submitted filename: Rebuttal letter.docx [file pone.0230126.s004.docx]

**Response to Reviewers**

Review Comments to the Author

Please use the space provided to explain your answers to the questions above. You may also include additional comments for the author, including concerns about dual publication, research ethics, or publication ethics. (Please upload your review as an attachment if it exceeds 20,000 characters)

**Reviewer #1**: Tan et al. have removed and analyzed existing data to address reviewers’ concerns about the original draft of their manuscript. Their existing findings and conclusions relating gene targeting (GT) efficiency to cell type (i.e. ESC, iPSC, and MEF) have been removed to focus on the mechanisms underlying GT at the mouse Myh9 locus in ESCs. In response to reviewers’ suggestions, the authors have also analyzed genome sequence elements such as CG content, SSR, and SINE to consider functional elements possibly contributing to the high GT efficiency, specifically at the immediate regions flanking exon 2, which serve as the homology arms. They present this analysis on other species for comparison as well as present sequence elements for their other targeting sequences at intron 2 and exon 3. They have also presented gel images in the figures to show the outcome of their PCR results. Finally, the authors performed correlation analysis between total length of homology arms and targeting efficiency to quantitatively demonstrate a relationship.

**Answer: All the authors thank the reviewer for appreciating the improvement of the manuscript.**

The major concern remaining with the currently revised manuscript is the relevance of the genomics sequence comparison with other species. All three reviewers raised this concern and recognized that without GT efficiency data from other species, the comparison of genomic features is meaningless. The cross-species comparison of the locus sequence similarity is not an effective method to convey the mechanism of the high GT efficiency. One cannot draw any association between genetic features to function when data on function is completely not presented. This effort is better applied to compare genomics features among the Myh9 locus with other mouse loci that have regularly been used for GT in mouse ESCs, such as Rosa26, HPRT, or Col1a1, where the GT efficiency has been reported by other groups (PMID: 25143803, PMID: 17183668, PMID: 16400644, PMID: 19141541). While it would be more scientifically rigorous to for the authors to perform these GT experiments at these other loci themselves in the same cell lines they have reported on in this manuscript, they can perform meta-analysis by drawing associations based on data previously published by other labs based on their targeting constructs.

**Answer: Thanks to the reviewer for this good suggestion! After serious consideration, we decide to remove the comparison of the Myh9 gene locus and the sequences used to create the construct targeting the Myh9 locus with the counterparts from other species. On the one hand, it more seems to be negative data; a functional confirmation of these genetic feature is lacking, on the other hand. According to the suggestion from this reviewer, in the new version, we collected related information about gene targeting at the Rosa26, HRPT, ColA1 loci suggested by the reviewer. The summarization and comparison of gene targeting events and genetic sequences at these loci with those at the Myh9 locus. These contents were described and presented in the new version. Though no common genetic characteristics could be identified among those four loci, a typical 6kb total length for the homologous arms, a 2:1 ratio for the length of the 5’ to 3’ homologous arm, similar and 50% GC-content for the 5’ and 3’ arms, seemed to have better performance, as further confirmed in our subsequent experiments.**

Despite this reviewer’s opinion that the species comparisons should not be performed and that the data do not support the authors’ conclusion, there are critical misinterpretations of the data as they are currently presented:

**Answer: Thanks a lot. Since the comparison and related data were removed, there was no this issue in the revised version.**

The methods section describes analyzing only the flanking 5’ and 3’ sequences of Myh9 exon 2 in all species. This is not presented in Figure 1A, which makes it rather misleading to think that Figures 1B, C, and D are based on Figure 1A. In Figure 1A, why is the orientation of the Myh9 loci reversed for the Pig and the Rabbit? This is confusing when the authors refer the right and left arm, rather than the 5’ and 3’ sequences. In Figure 1B, C, and D, the relationships are simply reflecting what is expected to be the relationship on a genome wide level, therefore one should not be surprised that mouse and rat bear such a high degree of similarity. Also, the algorithm for calculating percent identity and divergence should be clearly described for the reader.

**Answer: Thanks to the reviewer for this concern. Similarly, the part related to the comparison and analysis of the Myh9 locus and sequences with those of other species has been removed, the concern from the reviewer was not an issue.**

Figure 2: PCR data is missing for intron 2.

**Answer: Because a representative image for PCR identification of targeting events at the Myh9 intron2 site has been presented in our previous report [Liu T et al. PLos One, 2018], similar result was therefore omitted in this study.**

Figure 3 is mislabled as Figure 4.

**Answer: Thanks for the correction. All the figures have been reorganized and marked clearly.**

The targeting construct series can benefit the reader by listing properties of each construct to the left of each diagram, such as combined length of homology arms, the ratio of 5’:3’, etc. the length of each arm should also be displayed next to each line.

**Answer: Thanks for the good suggestion. Related information has been added and this figure has been modified.**

Figure 3C is basically Figure 3D, but it is not appropriate to apply this type of graphing to this data set. The authors should only present **scatter plots** in the style of Figure 3D.

**Answer: Thanks for the good suggestion. This graph has been modified in the new version.**

In Figure 3D, the authors perform a quantitative measure of the relationship between total length of homology arms and GT efficiency. They can test the significance of this correlation.

**Answer: Thanks for the suggestion. The test of the correlation significance (Represented by p<0.01) between the total length of homologous arms and the GT efficiency has been performed and presented in the revised version.**

The authors should also perform similar quantifiable analysis using the ratio of 5’:3’ lengths, GC content, SSR, and SINE of total, and individual homology arms. These analyses may provide some more insight into the mechanisms underlying the respective GT efficiencies.

**Answer: Thanks a lot for this good suggestion. The genetic feature analysis of the sequences used for creating different length homologous arms has been conducted, which is summarized and presented in the new version.**

Table 4. Why is only one repeat shown? Or is this the sum of 2 repeats? Authors should specify.

**Answer: Thanks for raising this concern. This point has been specified in the figure legends of the new version.**

Altogether, the manuscript as presented lack sufficient data to model a hypothesis, much less support a detailed, mechanistic understanding of why the Myh9 locus at exon 2 is such an efficient site for GT. The suggested, more in-depth analyses could bring this manuscript to a body of work suitable for publication in PLOS One.

**Answer: Thanks a lot for the comments. According to the comments and suggestions from this reviewer, the contents have been adjusted, the related information has been provided, and the figures have been modified, therefore, the manuscript should be greatly improved and reach to the requirement for the journal of PLoS One.**

**Reviewer #2**: The manuscript is greatly improved, and is much more focused on the underlying data and findings. The level of detail provided will allow others to potentially use the murine Myh9 locus as a site to further optimize gene targeting. My only remaining concern (which was shared by other initial reviewers) is the comparison of the murine Myh9 locus with that of other species. With zero gene targeting information for the Myh9 locus in these other species, this is just a distraction for the reader. The inbred mouse is an invaluable tool in biomedical research, and this manuscript should focus on the murine Myh9 locus without speculating about orthologous sequence and how that relates or does NOT relate to the gene targeting. Furthermore, it is misleading to assume that "No unique features and low sequence similarity are found in the Myh9 gene" when comparing to other Myh9 loci for which there is zero gene targeting information. Perhaps a better comparison would be to randomly sample other murine paralogs or similar length/structure loci to see if there is something unique to the murine Myh9 locus that affords such high gene targeting efficiency.

**Answer: Thanks to the reviewer for appreciating the improvement of this manuscript and providing other useful suggestions. We have removed the comparison of the Myh9 locus/Sequences from multiple species. Instead of these, we collected, organized and compared the targeting efficiency and the genetic features of several loci often used for site-specific integration, as also suggested by other reviewers. The new content is provided in the revised version.**

**Reviewer #3:** Tan et al have partially addressed some concerns issued on their manuscript, "Investigation of the molecular biology underlying the pronounced high gene targeting frequency at the Myh9 gene locus in mouse embryonic stem cells." The reasons for similar length of the homologous arms have been properly elucidated. And the further bioinformatic analysis have been performed. However, some issues still should be addressed or discussed to make the manuscript fit for publication in plos one.

**Answer: Thanks to the reviewer for appreciating the improvement of this manuscript and providing other useful comments.**

Comment-1
As mentioned before, the authors analyzed the locus of the Myh9 gene in multiple species. While there is no data showing the GT frequency in these species. Therefore, it’s hardly to reach the conclusion that low correlation with the high GT frequency at the Myh9 gene in term of chromosome position from the variable or conserved the locus Myh9 and its flanking genes.

**Answer: Thanks for raising this concern. We have deleted the comparison of the Myh9 locus/Sequences from multiple species. Instead of these, we collected, organized and compared the targeting efficiency and the genetic features of several loci often used for site-specific integration, as also suggested by other reviewers. The new content is provided in the revised version.**

Comment-2
The authors used the V6.5 ESCs which were derived from F1 hybrid mice, with 50% 129S6/SvEv and 50% C57BL/6 for gene targeting, and declared the constructs are isogenic to one of the two chromosomes, while there is no data shows the homology between 129/Sv and C57BL/6 in this loci. Is there evidence that HR is occurs only in the Myh9 loci of 1296/Sv? If not, maybe there were some homozygous clones for the targeted allele, which may result in bias for colony picking, as the authors mention, for homozygous knockout of Myh9 can cause abnormal proliferation and ESC morphology. If homology is low, is the targeting efficiency of the Myh9 loci in C57BL/6 as high as 129S/Sv?

**Answer: Thanks for raising this concern. The BAC DNA used as the template for obtaining the homologous arms is derived from a 129/Sv genetic background. Importantly, the blast of the whole sequences used for creating the homologous arms indicated that the sequences are100% matching with these from a C57BL/6 genetic background (using the NCBI/Blast program). In other words, the sequences used for creating the targeting constructs are isogenic to both chromosomes. Likewise, no homozygous ES clones were identified by either PCR or Southern Blot.**

Comment-3
Unformal writing of gene name such as line 40 “…Myh9 (Italic), line 170 “…MYH9” and Figure 1A. In Figure 3C, the units of vertical and horizontal axis should be provided. In Figure 3D, as targeting efficiency will not be lower than 0, it is more appropriate to set the minimum value of the vertical axis to 0.

**Answer: Thanks for these corrections. We have corrected all of them.**
