# Supplementary material for: Investigation of the molecular biology underlying the pronounced high gene targeting frequency at the Myh9 gene locus in mouse embryonic stem cells
Source: PLoS One. 2020 Mar 30;15(3):e0230126. doi: 10.1371/journal.pone.0230126 (PMC7105122; doi:10.1371/journal.pone.0230126)
Supplement: Attachment — Submitted filename: Response to reviewers.doc [file pone.0230126.s005.doc]

**Response to reviewers**

Reviewer #1: (No Response)

-Reviewer #2: The manuscript in its second revised form is greatly improved. The removal of speculative gene targeting of the Myh9 locus in other species from the main manuscript places focus on the actual work done. Supplementary Figure 1 is poor resolution (and completely unnecessary). While I understand that the multi-species Myh9 locus alignment was time consuming to generate, it really does not add anything of value to the manuscript.

**Answer：All the authors thank the reviewer for appreciating the improvement of the revised manuscript. The supplementary Figure 1 has been removed from this manuscript.**

-Figure 2C and 2D are the exact same data. there is no need to present the same data twice, just add a trend line to 2C (scale on 2D is nonsensical, -20% targeting efficiency?)

**Answer: Thanks for your suggestion. Fig 2D has been deleted, and a trend line has been incorporated into Figure 2C.**

-Throughout the paper the Myh9 introns and exons are capitalized and written both with and without a space before the number (e.g. Exon2, Exon 2, Intron2, Intron2, etc). Intent is obvious, but it should be at least consistent within the manuscript. Decision to capitalize intron and exon is questionable, but not a scientific issue.

**Answer: Thanks for your correction. We have used exon2 or intron2 instead of other ones in the whole manuscript to make sure the consistency.**

Reviewer #3: Tan et al. have removed the comparison of the Myh9 locus from multiple species, and according to reviewers’ suggestion, the authors compared the length of homologous arms and genomics elements (CG content, SSR, CpG islands, Line, SINE) of the Myh9 locus with other mouse loci (Rosa26, HPRT, and Col1a1) that have widely been used for GT to reveal characteristics potentially contributing to the high GT efficiency. They have also analyzed genomics features of the truncated homologous arms.

**Answer: All the authors thank the reviewer for appreciating the improvement of the manuscript.**

Comment-1 The author removed the comparison of the Myh9 locus from multiple species, however, retains the alignment of these sequences in Supplementary Figure 1, and it may more preferably to removed Supplementary Figure 1 together.

**Answer: Thanks a lot for your suggestion, Supplementary Figure 1 has been removed.**

Comment-2 Without GT efficiency data from other mouse loci with different ratio of homologous arms, it is inappropriate to infer 2:1 ratio for the length of the 5’ and 3’ homologous arms facilitate GT efficiency from the result of comparison the Myh9 locus with other loci.

**Answer: Thanks a lot for your consideration, we have amended this speculation for other loci.**

Comment-3 As other reviewer’ suggestion, Figure 3C is basically Figure 3D, the authors should only present Figure 3D and removed Figure 3C. The concern remaining in the Figure 3D, as targeting efficiency will not be lower than 0, it is more appropriate to set the minimum value of the vertical axis to 0.

**Answer: Thanks a lot for your suggestion. Fig 2C and Fig2D has been incorporated into one, e.g Fig2C in the revised version, your concern has also been solved.**

Comment-4 In Figure 2A, No.2 total length is 4.6 kb, and the right homologous arm of No.3 should be left aligned.

**Answer: Thanks a lot for your correction, this error for construct No2 has been amended, while the information for construct No3 was right, because we deleted the exon2 and beyond (an about 0.5kb) to obtain this left truncated form of 3’arm. A note for this has been added in the legend for Figure2A.**
